# Mimicking human riboflavin responsive neuromuscular disorders by silencing *flad‐1* gene in *C. elegans* : Alteration of vitamin transport and cholinergic transmission

**DOI:** 10.1002/iub.2553

**Published:** 2021-09-24

**Authors:** Piero Leone, Maria Tolomeo, Elisabetta Piancone, Pier Giorgio Puzzovio, Carla De Giorgi, Cesare Indiveri, Elia Di Schiavi, Maria Barile

**Affiliations:** ^1^ Department of Biosciences, Biotechnology, and Biopharmaceutics University of Bari Bari Italy; ^2^ Faculty of Medicine, Pharmacology and Experimental Therapeutics Unit, Institute for Drug Research, School of Pharmacy The Hebrew University of Jerusalem Jerusalem Israel; ^3^ Department DiBEST (Biologia, Ecologia, Scienze della Terra), Unit of Biochemistry and Molecular Biotechnology University of Calabria Arcavacata di Rende Italy; ^4^ Institute of Biosciences and Bioresources (IBBR) CNR Naples Italy

**Keywords:** *C. elegans*, ETFDH, *flad‐1*, LSMFLAD, *rft‐1*, riboflavin therapy, RNA interference

## Abstract

Riboflavin (Rf), or vitamin B2, is the precursor of FMN and FAD, redox cofactors of several dehydrogenases involved in energy metabolism, redox balance and other cell regulatory processes. FAD synthase, coded by *FLAD1* gene in humans, is the last enzyme in the pathway converting Rf into FAD. Mutations in *FLAD1* gene are responsible for neuromuscular disorders, in some cases treatable with Rf. In order to mimic these disorders, the *Caenorhabditis elegans* (*C. elegans*) gene orthologue of *FLAD1* (*flad‐1*) was silenced in a model strain hypersensitive to RNA interference in nervous system. Silencing *flad‐1* resulted in a significant decrease in total flavin content, paralleled by a decrease in the level of the FAD‐dependent ETFDH protein and by a secondary transcriptional down‐regulation of the Rf transporter 1 (*rft‐*1) possibly responsible for the total flavin content decrease. Conversely an increased ETFDH mRNA content was found. These biochemical changes were accompanied by significant phenotypical changes, including impairments of fertility and locomotion due to altered cholinergic transmission, as indicated by the increased sensitivity to aldicarb. A proposal is made that neuronal acetylcholine production/release is affected by alteration of Rf homeostasis. Rf supplementation restored flavin content, increased *rft‐1* transcript levels and eliminated locomotion defects. In this aspect, *C. elegans* could provide a low‐cost animal model to elucidate the molecular rationale for Rf therapy in human Rf responsive neuromuscular disorders and to screen other molecules with therapeutic potential.

## 1 INTRODUCTION

Riboflavin (Rf) or vitamin B2 is an essential molecule for humans, who cannot synthesise it, and therefore, must obtain the vitamin from intestinal absorption. This process is mediated by the recently characterized Rf transporters (hRFVTs), belonging to the SLC52 family.^1^, ^2^ Conversion of intracellular Rf into the flavin cofactors FMN (flavin mononucleotide) and FAD (flavin adenine dinucleotide) occurs via the sequential action of Rf kinase (RFK, EC 2.7.1.26), which transfers a phosphoryl group from ATP to Rf to form FMN, and of FAD synthase (FADS, or ATP:FMN adenylyl transferase, EC 2.7.7.2), responsible for FMN adenylation to FAD.

As far as the latter enzyme is concerned, in mammalian cells it exists in different protein variants, localised in different sub‐cellular compartments,^2^, ^3^ as generated by alternative splicing of *FLAD1* gene. The most abundant and best characterised FADS isoform, namely isoform 2, consists of two fused domains, with the C‐terminus one being *per se* able to catalyse FAD synthesis and delivery to nascent cognate apo‐flavoproteins in a redox sensitive way.^4^, ^5^, ^6^ Conversely, the N‐terminal domain of hFADS2 performs a Co^2+^ dependent hydrolytic activity towards FAD and NADH (EC 3.6.1.18), maybe responding to changes in the redox state of specific cysteines.^5^, ^7^

All these findings appear to have interesting—although not yet elucidated in detail—roles in the mechanisms controlling intracellular homeostasis of flavin cofactors, which is expected to control the maintenance of cellular flavoproteome, which consists of the product of at least 90 genes in humans, 84% of which use FAD as cofactor. A number of fundamental metabolic pathways and regulatory processes, among which the beta‐oxidation of fatty acids and the functionality of the mitochondrial respiratory chain depend on flavoenzymes and, therefore, require the constant supply of the two redox cofactors FMN and FAD (for comprehensive reviews see References 2, 8, 9). Thus, it is not surprising that flavoenzyme deficiency and deregulation of flavin homeostasis have been linked to several human diseases, such as neuromuscular and neurological disorders.^10^, ^11^, ^12^, ^13^

Here, we focus our attention on metabolic disorders which respond to high doses of Rf as some cases of MADD (Multiple Acyl‐CoA Dehydrogenase Deficiency, OMIM #231680), a rare autosomal inherited disease mainly characterized by organic aciduria and lipid droplets accumulation in skeletal muscle due to alteration of beta‐oxidation, and respiratory‐chain deficiency. The majority, but not all, Rf responsive MADD patients presented mutations in the gene coding for the FAD‐dependent ETF:QO (ETF:ubiquinone oxidoreductase or ETFDH, ETF dehydrogenase EC 1.5.5.1), embedded in the inner mitochondrial membrane, working as a funnel of electrons deriving from a number of Acyl‐CoAs, aminoacids, and choline.^2^, ^10^, ^12^, ^14^ Only recently a severe form of MADD (sometimes reverted by Rf treatment) has been associated to alteration of the *FLAD1* gene; it is now named LSMFLAD, which stands for Lipid Storage Myopathy due to FADS Deficiency (OMIM #255100).^12^, ^13^ Mutations of *FLAD1* gene severely affect muscular and cardiac functionality in humans.^11^, ^12^, ^13^, ^15^

Other genes correlated to Rf homeostasis are causative of metabolic neuromuscular disorders (for exhaustive reviews see References 12, 16 and references therein). Noteworthy is the Brown‐Vialetto‐Van Laere Syndrome (BVVLS, also known as Rf Transporter Deficiency, RTD), a rare neurological disease in which the functionality of either SLC52A3 (BVVLS1, OMIM #211530) or SLC52A2 (BVVLS2, OMIM #614707) is altered.^17^, ^18^

The molecular rationale for Rf treatment in humans, as well as a number of fundamental processes involved in ensuring cellular FAD homeostasis are still far to be elucidated and claim further research. In order to mimic at the organism level the molecular defects underlying Rf responsive human pathologies, we introduced, long ago, two model systems: *Saccharomyces cerevisiae* strains lacking of the mitochondrial FAD transporter gene, namely *FLX1*,^19^, ^20^ and *Caenorhabditis elegans* (*C. elegans*) in which RNA interference was used to silence the single copy of *flad‐1* gene (R53.1, WBGene00011271, Wormbase).^21^

Focusing on *C. elegans*, we found that *flad‐1* gene is the orthologue of the human gene coding for FADS and generates 2 different protein isoforms by trans‐splicing mechanisms. The two isoforms show a 37% identity and 55% similarity to the human FADS1.^21^

In *flad‐1* silenced animals flavin homeostasis was altered with impact on mitochondrial bioenergetics, ATP and ROS productions, resulting in a profound protein homeostasis alteration and alteration of complex behavioral patterns, as fertility, locomotion,^21^ and lifespan.^22^ The hypothesis that *flad‐1* silencing might affect cholinergic transmission of worms was also brought forward.^21^

Among the potential “cognates” of FAD synthase, of pivotal importance in bioenergetics, the product of human *ETFDH* gene associated to MADD deserves deep attention. Interestingly enough, most of the residues mutated in human *ETFDH* gene in MADD are conserved in the *C. elegans* orthologue *let‐721* (WBGene00002855),^23^ whose only product LET‐721 (CE29662, Wormbase), shows 63% identity and 77% similarity (performed by Blastp) with the human corresponding mitochondrial precursor protein (Q16134‐1, in Uniprot). Low levels of flavin cofactors may cause ETFDH instability or altered folding, in turn triggering mitochondrial protein unfolding response (mtUPR) in mammals.^24^, ^25^

In the frame of identification of the worm genes coding for Rf translocators, named *rft‐1* and *rft‐2*, an alternative model was created in Said laboratory. Silencing *rft* genes resulted in a profound alteration of worm fertility and inhibition of feeding by light.^26^, ^27^

In this article, the possible involvement of *flad‐1* gene in neuromuscular function in *C. elegans* has been experimentally approached and the molecular rationale for Rf therapy in MADD/LSMFLAD further deal with. To this aim *flad‐1* gene has been silenced via transient interference in two different genetic backgrounds. Indeed, both interfered strains chosen for our investigations show locomotion defects and other molecular and functional alterations. Among these, expression changes of the Rf translocator have been revealed. On the basis of all the experimental data described, a proposal is made that altering Rf metabolism impacts on neuronal cholinergic trophism.

## 2 EXPERIMENTAL PROCEDURES

### 2.1 Materials

All reagents and enzymes for biochemical analysis were from Sigma‐Aldrich (USA). Reagents for RNA extraction and retro‐transcription (Trizol Reagent, SuperScript III First‐Strand System for RT‐RNA), chemically competent *E. coli* cells (Shot TOP10F') were purchased from Invitrogen (USA). RNA purification was performed with RQ1 RNAse‐Free DNAse (Promega). Reagents used for animal cultures were from Oxoid (UK). The dye reagent for protein assay was from Bio‐Rad (USA). Solvents and salts used for HPLC were from J. T. Baker (USA). Other chemicals for electrophoresis were from either Merck (Germany) or Bio‐Rad (USA).

### 2.2 *C. elegans* culture conditions

*C. elegans* N2 (wild type) and *rrf‐3 (pk1426)* strains were maintained on Nematode Growth Medium (NGM) agar plates (51.72 mM NaCl, 1.7% bacto‐agar, 0.25% bacto‐peptone, 0.0005% cholesterol, 0.5 mM CaCl_2_, 1 mM MgSO_4_, 4 mM potassium phosphate pH 6) seeded with *E. coli*, OP50 strain. The *rrf‐3 (pk1426)* strain was provided by the Caenorhabditis Genetic Center. The animals were incubated at 22°C and observed through a Motic SMZ‐171 stereomicroscope. The life cycle of *C. elegans* is comprised of the embryonic stage, four larval stages (L1‐L4) and adulthood. The end of each larval stage is marked with a moult where a new, stage‐specific cuticle is synthesized and the old one is shed. Each stage is easily identified by the relative size of larvae.^28^

### 2.3 RNA interference

RNA interference (RNAi) was brought about by feeding.^29^ Empty vector L4440 was purchased from Addgene (http://www.addgene.org) and used to transform HT115 *E. coli* cells. These clones were used as control for the RNAi interfering clones. RNAi transfected bacterial clones II‐7 G03/R53.1 were obtained from Geneservice Ltd (UK). One colony was picked from LB plates containing 50 μg/ml ampicillin, transferred into 2 ml LB broth containing 50 μg/ml ampicillin, and grown overnight at 37°C with shaking. The following day the bacterial culture was diluted 1:4 and allowed to grow one more hour, then the culture was supplemented with 1 mM IPTG (isopropyl β‐d‐1‐thiogalactopyranoside) and plated onto NGM containing 50 μg/ml ampicillin and 1 mM IPTG. After 3 h of induction, L4 animals were transferred to the RNAi plates and incubated for 3 days at 20°C. After the third day, this protocol has been repeated to produce a stronger interference. For some experiments, culture plates were supplemented with 5 μM Rf, FMN, and FAD, respectively.

### 2.4 Semi‐quantitative RT‐PCR

Animals were collected from plates with M9 buffer (22 mM KH_2_PO_4_, 42 mM Na_2_HPO_4_, 83 mM NaCl, 0.1 mM MgSO_4_), and lysed by thermal shock. Total RNA was extracted using Trizol Reagent (Invitrogen) and treated with RQ1 RNAse‐Free DNAse (Promega). RNA concentration was measured at 260 nm. Total cDNA was prepared from 700 ng DNase‐treated RNA using SuperScript III First‐Strand System for RT‐PCR (Invitrogen). Random hexamers were used as primers for the overall cDNA synthesis. PCR was performed using Taq DNA Polymerase with MgCl_2_ Buffer (Sigma) according to the manufacturer's instructions using gene‐specific primers. RT‐PCR products were separated through electrophoresis on ethidium bromide‐containing 1.2% agarose gel. Gene expression level was analysed through a semi‐quantitative reverse transcription‐PCR technique, using *ama‐1* for expression level normalization of the target genes. Images and quantification were obtained with a ChemiDoc™ MP Imaging System (BIO‐RAD). Primers were designed on the basis of sequences retrieved from *C. elegans* database (Wormbase, http://www.wormbase.org) and are reported in Table S1.

### 2.5 Egg‐production assay

Before the experiment, adult hermaphrodite worms were synchronized by placing one gravid adult on an NGM plate and treating it with a NaOH/Bleach 1 N solution. This allowed the expulsion of eggs in the same embryonic stage. Eggs were allowed to hatch and grow until reaching gravid adult stage. Ten gravid adults have been placed on 10 NGM plates on which either 200 μL of H_2_O or 0.1 mM levamisole were added. Laid eggs were counted 5 h after transfer of the animals.

### 2.6 Chemosensory assay

A 5 cm Petri plate was divided in three areas. A 30 μl aliquot of OP50 *E. coli* liquid culture was applied and spread into a section of the plate as attractant whereas another 30 μl aliquot of 0.1% SDS was applied into the opposite section as repellent as described in.^21^ Where indicated, plates for the assay were supplemented with 5 μM Rf, FMN, and FAD, 0.75 mM levamisole, 1 mM aldicarb, 0.5 mM acetylcholine, respectively. Ten animals for each condition were placed at the center of the plate and the number of animals that reached the attractant area every 15 min, for a total of 2 h, was measured under a dissecting microscope.

### 2.7 Quantitation of flavins

Animals were harvested, washed in M9 buffer, suspended in NSB buffer (0.3% ethanolamine, 2 mM EDTA, 0.1 M PMSF, 5 mM DTT, ×1 protease inhibitor) and lysed by thermal shock. The lysate was centrifuged at 13,000×*g* for 1 min to remove cuticle and nuclei. Rf, FMN, and FAD content of the supernatant was measured in aliquots (5–80 μl) of a neutralized perchloric extract by means of HPLC (Gilson HPLC system including a model 306 pump and a model 307 pump equipped with a FP‐2020 Plus Jasco Fluorescence detector and Unipoint system software), essentially as previously described.^19^, ^20^

### 2.8 Western blotting

SDS‐PAGE separated proteins were electro‐transferred onto a PVDF membrane using a transblot semidry electrophoretic transfer cell (Sigma‐Aldrich). The immobilized proteins were incubated overnight with a 3000‐fold dilution of a polyclonal antiserum against FADS or against HSP‐60, or with a 5000‐fold diluted antibody against ETFDH. As protein marker, a mouse monoclonal anti‐GAPDH antibody (1:1000 dilution) was used. The bound antibodies were visualized with the aid of secondary anti‐rabbit or anti‐mouse IgG antibodies conjugated with peroxidase (1:2500 dilution).

### 2.9 Statistical analysis

Differences between RNAi‐treated and control animals were analyzed for statistical significance by using Student's *t*‐test.

## 3 RESULTS

### 3.1 Silencing *flad‐1* gene in *rrf‐3 (pk1426)* strain: ETFDH changes, mtUPR, and transcriptional changes

The concept that alteration of mitochondrial FAD supply in *C. elegans* profoundly affects mitochondrial respiratory chain activity, ATP production, redox balance, protein homeostasis, locomotion and egg‐laying was initially proposed in our laboratory by investigating the effect of transiently silencing *flad‐1* gene in a wild type N2 strain.^21^

The present investigation is aimed at enlarging the spectrum of information concerning the biochemical and functional consequences of lowering FAD cofactor levels in *C. elegans*, by silencing *flad‐1* gene, making use of *rrf‐3 (pk1426)* strain. This strain is characterized by the loss of function of a putative RNA‐directed RNA polymerase (RdRP) of *C. elegans* (RRF‐3, CE45624, Wormbase), resulting in a substantial enhancement of sensitivity to RNAi in diverse tissues. This is particularly striking in the nervous system, since neurons are generally refractory to RNAi in a wild type N2 genetic background.^30^ Indeed, *flad‐1* silencing in a *rrf‐3 (pk1426)* strain causes a slower growth of the animals,^31^ which is not observed when silencing a wild type N2 strain.^32^

**FIGURE 1:**
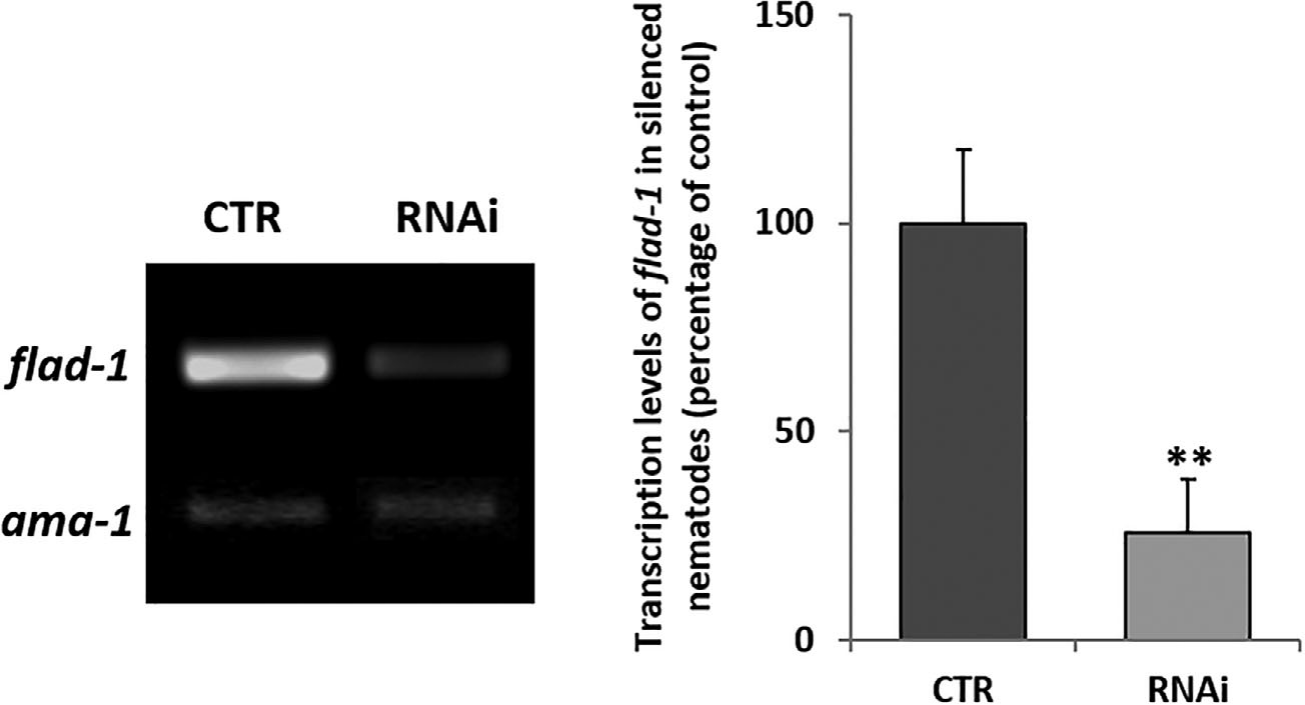
Silencing of *flad‐1* gene. *flad‐1* transcript levels in silenced (RNAi) versus control (CTR) *rrf‐3 (pk1426) C. elegans* were measured by RT‐PCR by using primers reported in Table S1, and referred to *ama‐1* transcripts used as a control. In the histogram panel each value is the mean ± SD of three different determinations (**p ≤ 0.01)

**FIGURE 2:**
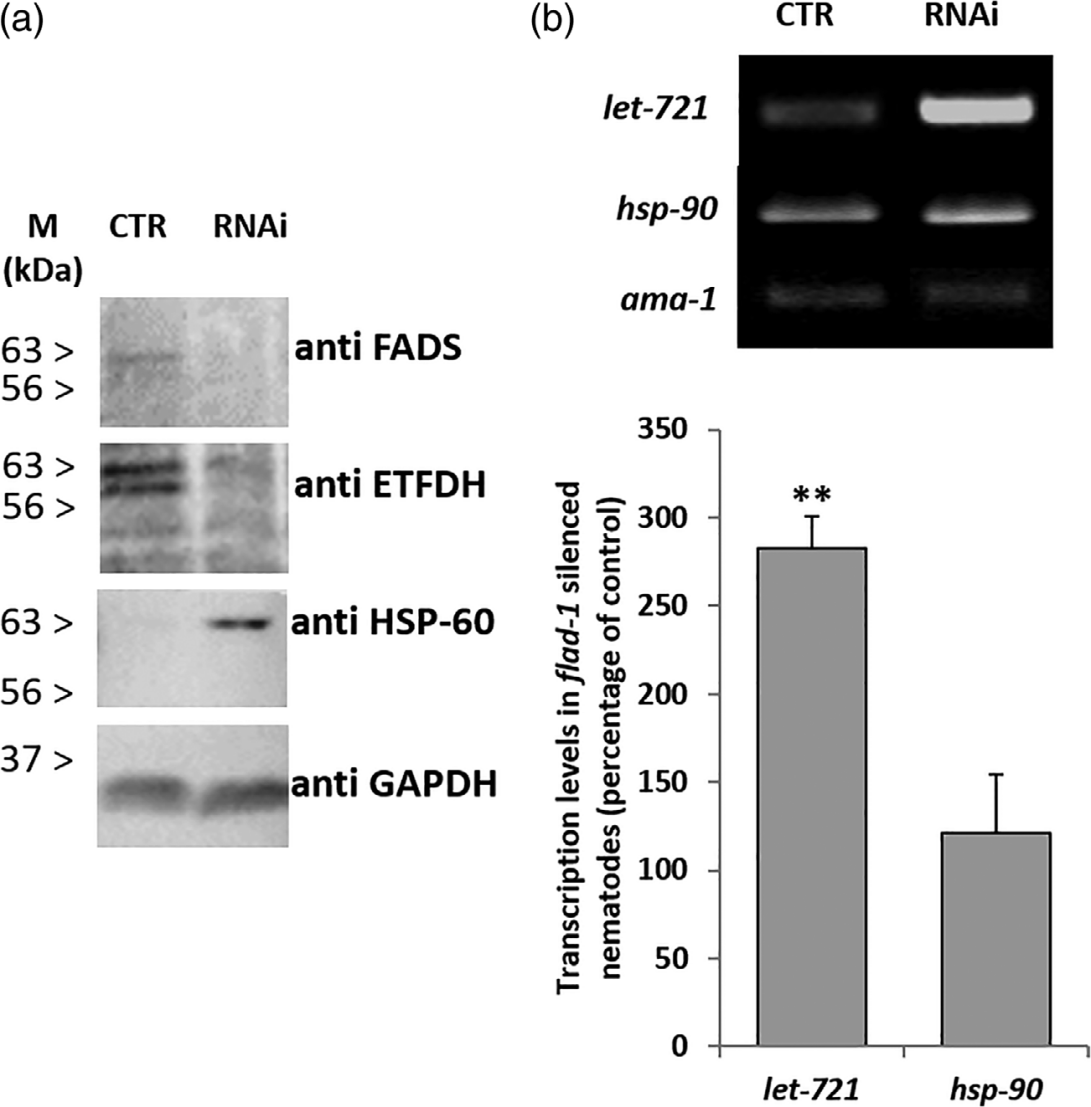
Protein and transcript levels. (A) FADS, ETFDH, or HSP‐60 proteins were detected in silenced *rrf‐3 (pk1426)* or in control animals by Western Blotting analysis performed as described in Experimental Procedures. GAPDH (or GPD‐1, CE02343 in Wormbase) bands are used as quantitative controls. The blotting in the figure is representative of two different analyses. (B) *let‐721* and *hsp‐90* transcript levels were measured by RT‐PCR by using primers reported in Table S1, and referred to *ama‐1* transcripts used as a control. In the histogram panel, each value is the mean ± SD of three different determinations (**p ≤ 0.01)

As shown in Figure 1, the silencing procedure resulted in a significant decrease in the overall *flad‐1* transcriptional level (75% as assessed by semi‐quantitative RT‐PCR). An even more significant decrease was observed in the amount of the protein bands detectable by Western Blotting with an anti‐FADS antiserum (Figure 2A).

To evaluate whether decreased FADS expression level might affect the mitochondrial flavoproteome, ETFDH levels were also detected in extracts from silenced animals and compared to non‐silenced animals. A significant decrease of protein amount was detectable in the silenced organisms and ETFDH decrease was accompanied by an increase of a band corresponding to the mitochondrial chaperonin HSP‐60 (CE27244, Wormbase) (Figure 2A). This is in agreement with the proposed mitochondrial response to protein unfolding (mtUPR), triggered by cofactor scarcity.^25^ It also should be noted that a significant increase in the *let‐721* transcript level was observed in the silenced strain, maybe responding to the nuclear stress status (Figure 2B).

We can hypothesise a sort of compensatory transcriptional effect aimed at repristinating the decreased EFTDH protein level, coordinated by the mtUPR. Conversely, a reticulum UPR seemed not to occur under these conditions, as proven by the unchanged transcript levels of *hsp‐90* (WBGene00000915) coding for HSP‐90 (CE05441, Wormbase) protein (Figure 2B).

A different response to an altered homeostasis of flavin cofactors was observed in the wild type N2 strain,^21^ where neither HSP‐60 protein, nor *let‐721* transcript increased (Figure S1B). Therefore, the response mechanism seemed to be active only in the more “susceptible” *rrf‐3 (pk1426)* genetic background. Moreover, the transcriptional stimulus operating in *rrf‐3 (pk1426)* strain seemed to be ETFDH‐specific, since no changes in transcript levels of genes coding for other flavoproteins, namely succinate dehydrogenase *sdha‐1* (WBGene00015391), lysine demethylase *lsd‐1* (WBGene00011615) (Figure S1A) were detected.

Based on these observations, *C. elegans* carrying decreased level of *flad‐1* products seemed to be a good novel model for mimicking human MADD or—more appropriately—LSMFLAD.

### 3.2 Phenotypical consequences of *flad‐1* silencing: Alteration of cholinergic transmission

*flad‐1* (RNAi) silenced animals are normal in appearance, size and colour, as well as entrance and exit from the dauer stage do not differ from control animals.

**FIGURE 3:**
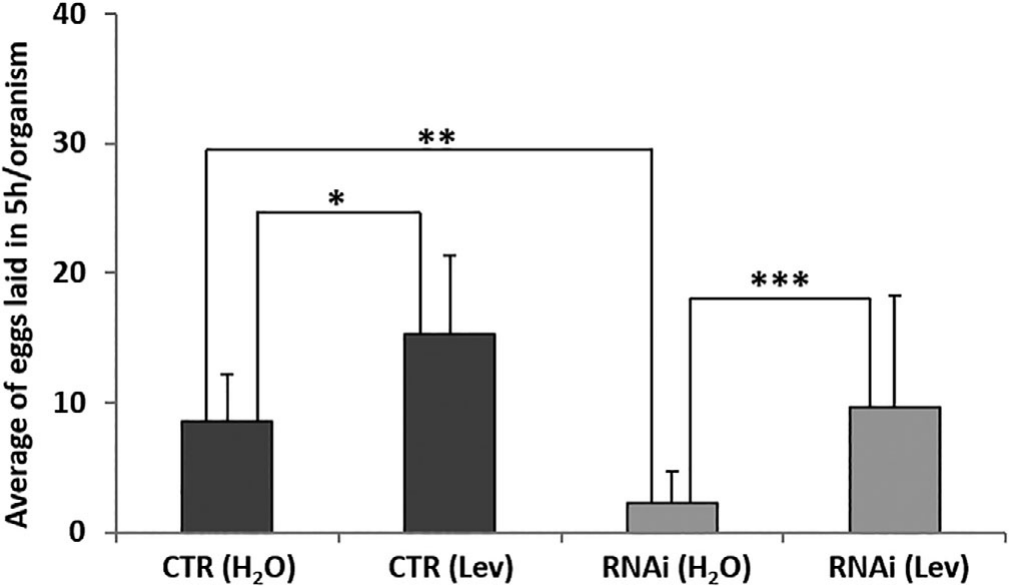
Egg production defects. Egg production assay was performed on 10 gravid adults as described in Experimental Procedures. The effect of 0.1 mM levamisole (Lev) addition, to both control and *flad‐1* silenced *rrf‐3 (pk1426)* animals, was measured and reported in histograms. Each value is the mean ± SD of three different determinations (*p ≤ 0.05; **p ≤ 0.01; ***p ≤ 0.001)

As a consequence of decrease in ETFDH levels, we expect an altered ability to utilise fatty acid, that is, to perform mitochondrial beta‐oxidation. A phenotypical aspect correlated to altered beta‐oxidation in *C. elegans* is a defect in fertility.^23^, ^27^ Consistently, in *flad‐1* silenced animals we registered an impaired ability to lay eggs in both fed^21^ and starved conditions (Figure 3). To prove that the phenotypical defect induced by *flad‐1* silencing consists in eggs production, rather than in eggs deposition, we analysed the response of both control and interfered animals to levamisole (L(−)‐2,3,5,6‐tetrahydro‐6‐phenylimidazo[2,1‐b]thiazole), a stimulator of cholinergic muscular receptor. The interfered animals still respond to levamisole, which however did not completely rescue the phenotype. These data are fairly in good agreement with a bioenergetics impairment affecting production rather than deposition.

**FIGURE 4:**
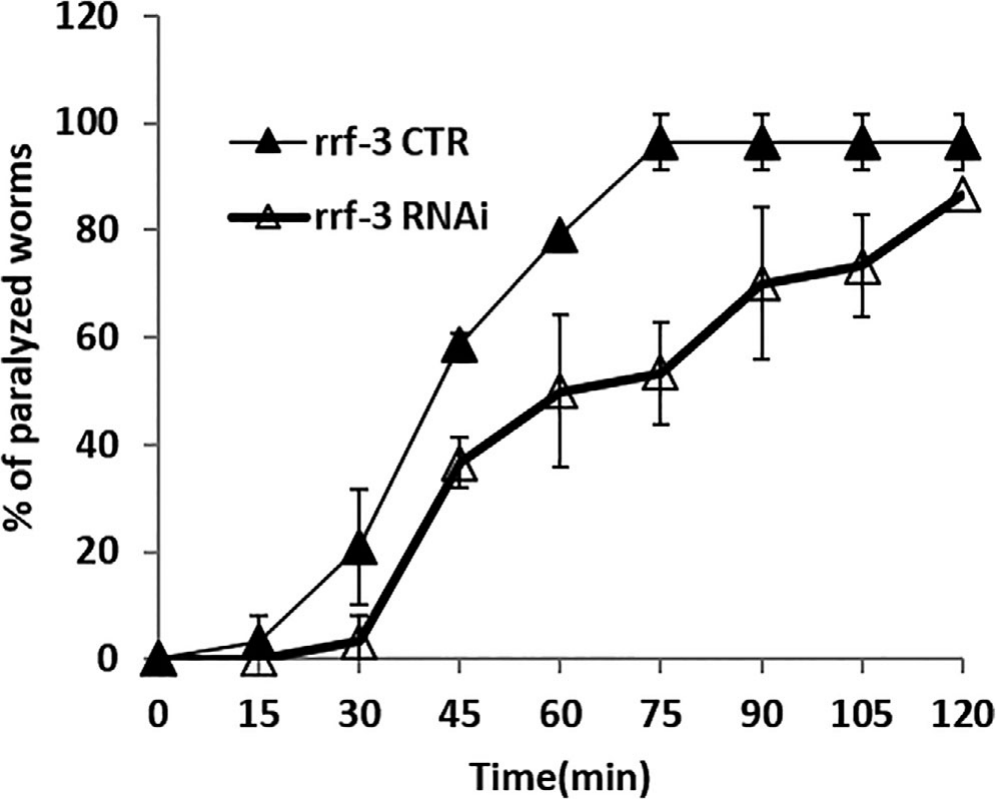
Sensitivity to aldicarb. Control (CTR) and silenced (RNAi) *rrf‐3 (pk1426)* animals were placed on plates containing 0.1 mM aldicarb and the percentage of paralyzed animals was recorded every 15 min. Each value is the mean ± SD of three different determinations

Other consequences of *flad‐1* silencing in the N2 animals,^21^ were a significant slow‐down of locomotion rate and a relative resistance to Aldicarb (2‐methyl‐2‐(methylthio)propanal O‐(N‐methylcarbamoyl)oxime), a known inhibitor of acetylcholinesterase.^33^ The *rrf‐3 (pk1426)* interfered animals were also partially resistant to aldicarb paralysis: 100% of the controls, but only about 50% of interfered animals were paralyzed, following 75 min observation (Figure 4). This confirms that locomotion defects are most likely connected with impairment of cholinergic signalling, either in the pre‐synaptic compartment (less Acetylcholine [ACh] released) or in the post‐synaptic compartment (lower responsivity to ACh).

**FIGURE 5:**
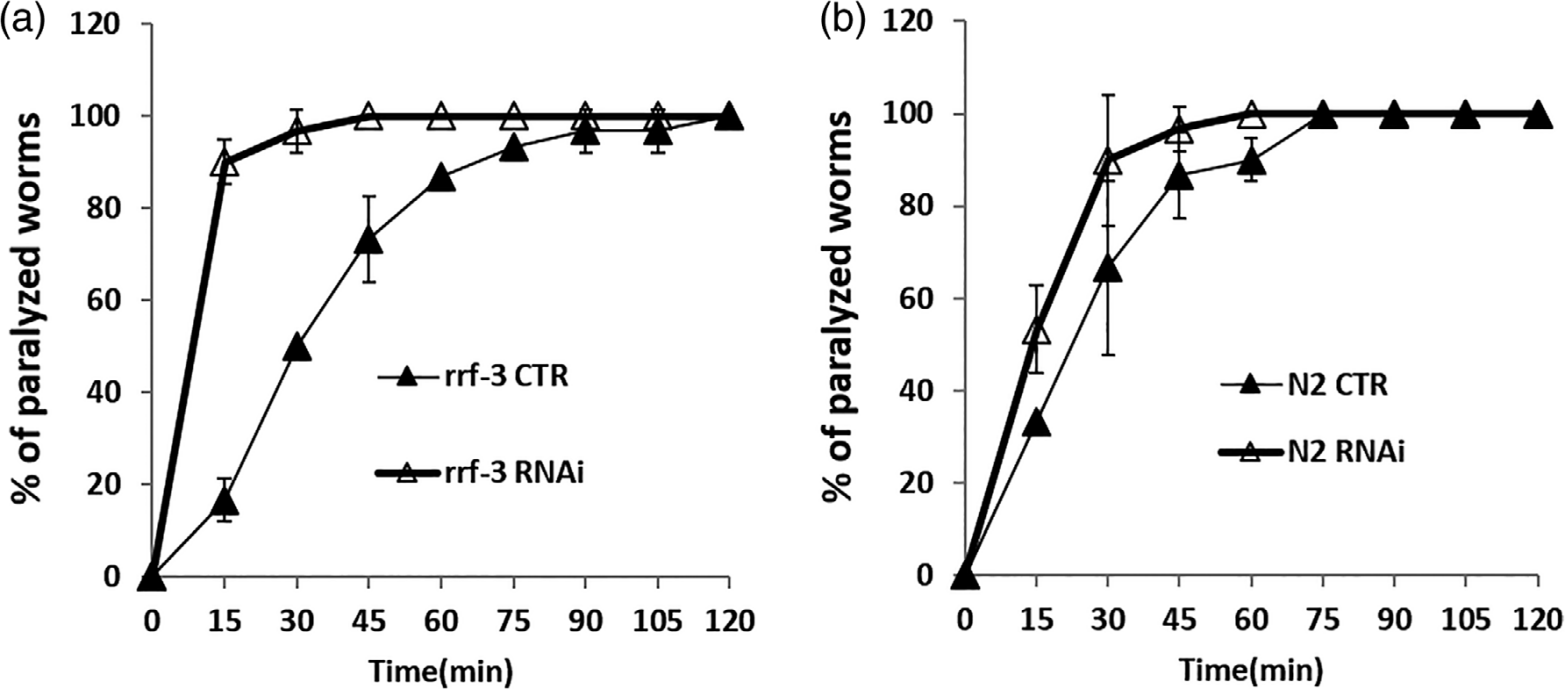
Sensitivity to levamisole. Control (CTR) and silenced (RNAi) *rrf‐3 (pk1426)* (A) or N2 (B) animals were placed on plates containing 0.75 mM levamisole and the percentage of paralyzed nematodes was recorded every 15 min. Each value is the mean ± SD of three different determinations

To dissect the role of post‐synaptic response, we tested the locomotion sensitivity to levamisole.^34^ A resistance to levamisole would suggest an alteration in post‐synaptic nAChRs (nicotinic Acetylcholine receptors), or putative downstream components at the NMJ (neuromuscular junction). Conversely, the percentage of paralyzed animals at each time was higher in silenced animals with respect to the controls. Almost 100% of interfered animals resulted paralyzed after 15 min observation, whereas the overall control animals were paralyzed after 90 min observation (Figure 5A). This increased sensitivity of silenced animals to levamisole is much less pronounced in the N2 silenced strain (Figure 5B). This suggests a defect in acetylcholine release in *rrf‐3 (pk1426)* interfered animals.

A similar levamisole hypersensitivity has been observed in other aldicarb resistant mutants, possibly reflecting a compensatory mechanism by which muscle cells compensate for decreased acetylcholine secretion by increasing their response to acetylcholine.^35^

Whatever the mechanism is, these observations lead us to confirm also in the *rrf‐3 (pk1426)* strain the occurrence of a cholinergic transmission alteration as a consequence of silencing *flad‐1* gene.

### 3.3 Rf treatment: Towards a molecular rationale involving *rft‐1*

Patients suffering for LSMFLAD and MADD can have beneficial effects from a therapy based on high doses of Rf; at the moment this is the best‐known therapy for these heterogeneous lipid storage myopathies. The molecular rationale of such effect is still under investigation. The most plausible hypothesis is based on an increased availability of FAD, which in turn could have a chaperoning effect on client apo‐flavoproteins.^25^

**FIGURE 6:**
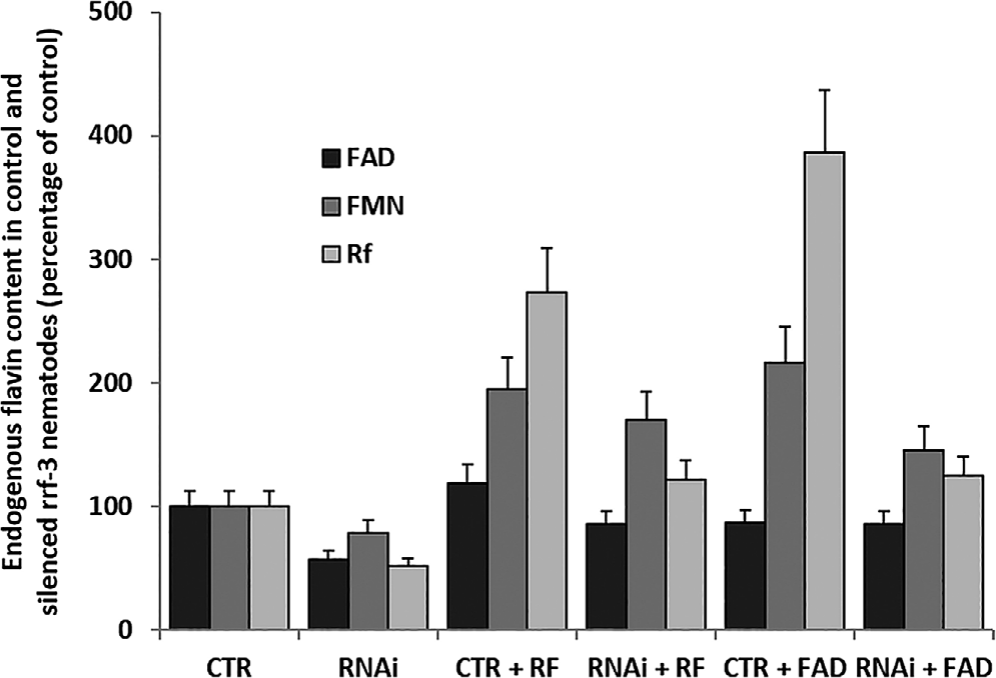
Quantitation of flavins. Deproteinized extracts were obtained from lysates of control and silenced (RNAi) *rrf‐3 (pk1426)* treated in the absence or the presence of 5 μM either Rf or FAD. Content of Rf, FMN, and FAD were measured by HPLC, as described in Experimental Procedures. Each value, as reported in the Table S2, is the mean ± SD of three different determinations

Therefore, we measured the levels of flavin cofactors by HPLC in deproteinized extracts from both control and silenced *rrf‐3 (pk1426)* animals (Figure 6). As expected, a significant decrease of FAD was observed (about 40%) in silenced animals; quite surprisingly, both FMN and Rf contents were concomitantly decreased (about 20% and 50%, respectively). To explain these findings, we hypothesized an impaired ability to take up the vitamin. Treatment of animals with 5 μM Rf or FAD during the silencing procedure, completely restored flavin levels.

To validate the hypothesis that the concomitant decrease of the three flavins is actually due to a reduced ability to transport the vitamin, we pointed our attention on *C. elegans* Rf translocators.^27^ Searching for the molecular rationale of neuronal phenotype, we decided to follow the expression levels of *rft‐1* gene (WBGene00044637), proven to be expressed in a small subset of neuronal support cells along the entire length of the animal and encoding a single protein isoform (RFT‐1, CE47043, Wormbase) of 427 aa.

**FIGURE 7:**
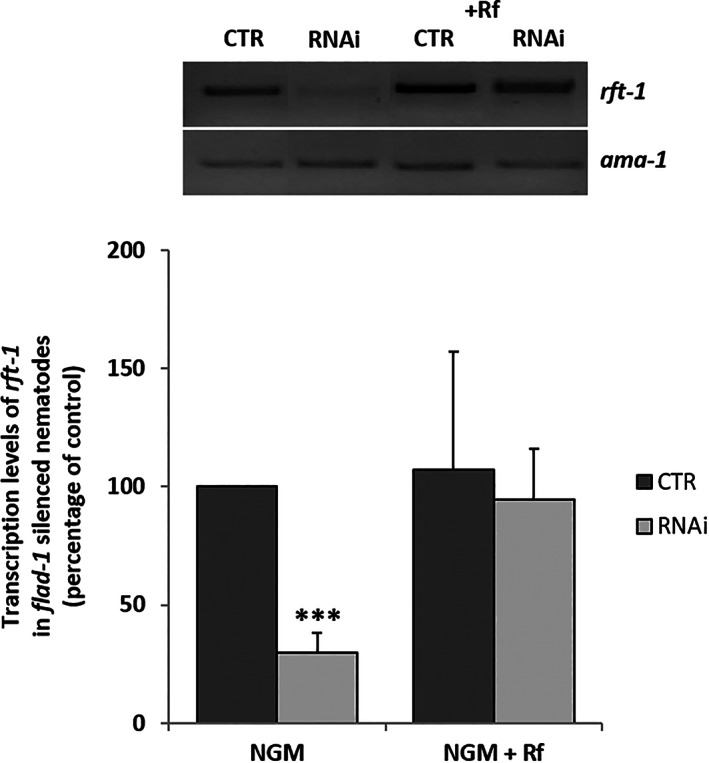
*rft‐1* transcript levels. Control (CTR) and silenced (RNAi) *rrf‐3 (pk1426)* animals were raised in the absence (NGM) or in the presence of 5 μM Rf (NGM + Rf). *rft‐1* transcript levels were measured by RT‐PCR by using primers reported in Table S1, and referred to *ama‐1* transcripts used as a control. In the histogram panel, each value is the mean ± SD of three different determinations (***p ≤ 0.001)

In *flad‐1* silenced animals, *rft‐1* transcript levels were significantly decreased (about 70%) with respect to the control (Figure 7). Treatment with Rf restored the transcript levels to their normal values.

We also checked in *flad‐1* silenced animals the expression levels of the intermediate component of the flavin cofactor biosynthetic pathway, that is Rf kinase (EC 2.7.1.26) coded by worm R10H10.6 gene (WBGene00011224), here indicated as *rfk‐1*, whose mutant tm7119 (https://shigen.nig.ac.jp/c.elegans/mutants/index.xhtml) is sterile or lethal. Differently from Rf transporter transcript, no change of *rfk‐1* transcript was detected (Figure S1C). Similar results were obtained with N2 strains (Figure S1D).

This is the first evidence in *C. elegans* model, that *flad‐1* silencing (or *ETFDH* gene or protein impairment), causes an alteration of Rf transport efficiency and more importantly, that this transcriptional derangement is Rf responsive.

### 3.4 Rf therapy: Effect on locomotion defects

**FIGURE 8:**
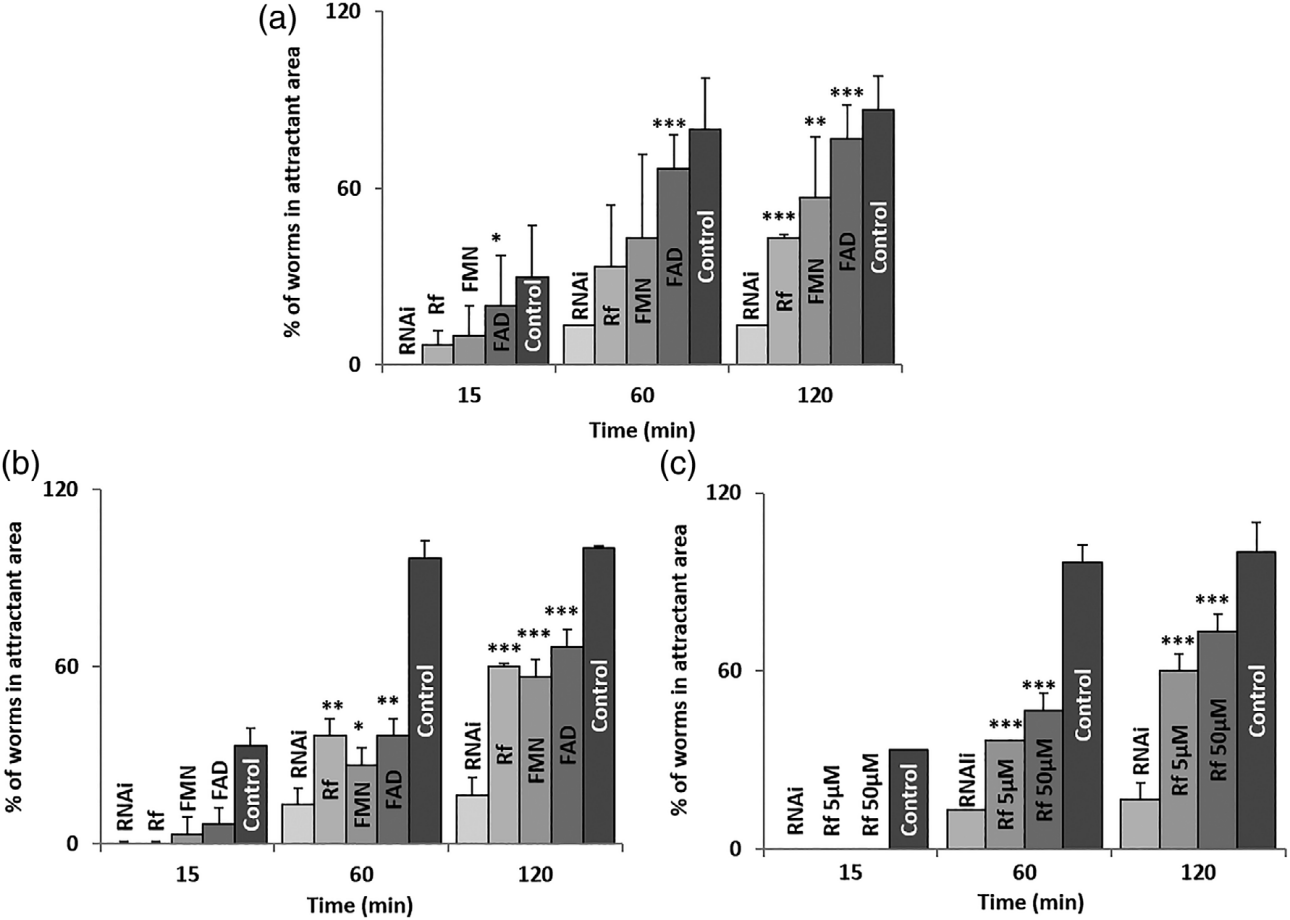
Rf responsiveness of defects in locomotion behavior induced by *flad‐1* silencing. Chemosensory assay was carried out on 10 animals per condition as described in Experimental Procedures. The percentage of nematodes in attractant area was recorded at different time points. Where indicated, 5 μM Rf, FMN, or FAD was added to the NMG plates during silencing process, as pre‐treatment (A), or in the experimental plates, as co‐treatment (B). Dose‐dependency was also tested (C). Each value is the mean ± SD of three determinations (*p ≤ 0.05; **p ≤ 0.01; ***p ≤ 0.001; referred to RNAi)

Another consequence of *flad‐1* silencing in *rrf‐3 (pk1426)* strain is the reduced ability of silenced animals to reach an attracting area. A typical experiment, performed essentially as in,^21^ is reported in Figure 8.

At different time points, a decreased fraction of silenced animals versus control can reach the attractant compound. The observed locomotion defects are prevented by growing *rrf‐3 (pk1426)* silenced animals in the presence of Rf. FMN, and especially FAD, are also effective in increasing the number of silenced individuals that can reach the attractant area at each time. The fraction of flavin‐treated RNAi worms almost reached the value of control individuals following 120 min locomotion (Figure 8A).

Similar results can be obtained if either Rf or flavin cofactors are added in the experimental plate during the time of observation (Figure 8B). Locomotion phenotype resulted ameliorated to the same extent if 0.5 mM acetylcholine was added in the experimental plate (data not shown). The last result is in agreement with the cholinergic pre‐synaptic phenotype we suspected.

The beneficial effects of flavin are dose‐dependent, as demonstrated by the slight, but significant increase from 5 to 50 μM of Rf concentration, from 60% to 73%, respectively (Figure 8C).

The molecular mechanisms that allow phenotypical restoration and adaptation of *rft‐1* transcription is still not known, but this effect could explain Rf responsiveness in LSMFLAD and MADD patients.

## 4 DISCUSSION

Since the discovery of *FLAD1* as a causing gene for the human severe mitochondrial myopathy,^36^ first classified as MADD, and recently named LSMFLAD,^12^, ^13^ the number of patients is rapidly increasing (see References 11, 12, 15, 16 for recent reviews). Unfortunately, not all patients respond to Rf therapy and this calls for further research manly devoted to find alternative pharmacological strategies. In this context, *flad‐1* silenced animals seemed to us a good promising low‐cost model for fundamental research and for novel drug screening.

Flavoenzyme derangement, ATP shortage and redox balance impairment, accompanied by infertility and alteration of locomotion behaviour, could make *flad‐1* silenced *C. elegans* animals a model system for studying human neuromuscular pathologies with alteration in flavin homeostasis/flavoenzyme biogenesis, as suggested in the past,^21^, ^37^ and further confirmed by experiments presented in this article.

The primary defect of beta‐oxidation pathway in MADD patients is due to gene defects leading to alteration of ETFDH stability/folding, somehow related to FAD availability.^13^, ^25^ Consistently, ETFDH levels are secondarily decreased in LSMFLAD patients.^36^, ^38^, ^39^ A secondary ETFDH derangement has been observed also in our worm model. ETFDH‐defective mitochondria may generate ROS and may give raise to mtUPR.^25^, ^40^ In *rrf‐3 (pk1426)* strain we, indeed, registered a mtUPR by checking HSP‐60 levels,^41^ in immuno‐blotting experiments carried out on worm extracts. These observations further validate our *C. elegans* model. We also registered an increase in *let‐721* transcripts. At the moment, there is no clear demonstration that ETFDH expression can be transcriptionally regulated by mtUPR signalling pathway. In addition, a search in the upstream region of the human *ETFDH* gene carried out with the Jaspar software (http://jaspar.genereg.net/) returns a responsive element for the transcription factor SOX12, performing a high degree of identity with the yeast Rox1 protein, crucial for this type of proteostasis stress.^41^

Additionally, in both N2 and in *rrf‐3 (pk1426) flad‐1* interfered strains, we found a significant decrease of Rf translocator transcript, logically responsible for the significant decrease in the intracellular concentration of the three flavins that we registered by HPLC in *C. elegans* extracts. RFVT proteins are normally expressed at low level in animal cells and they are quite difficult to solubilise^42^: this could be the reason why RFTs' level changes have been missed in the list of the *C. elegans* proteins responding to the stress derived by flavin unavailability, by the proteomic approach used in N2 strain.^21^ Treatments of animals with exogenous flavins, not only increased the *rft‐1* transcript levels, but also restored the secondary deficiency induced by *flad‐1* silencing. These are the first evidences, in *C. elegans* model, that *flad‐1* silencing (or *ETFDH* gene or protein impairment) causes an alteration of Rf transport efficiency, and more importantly, that this derangement is Rf responsive. We actually do not know whether *rft‐1* transcript level regulation is directly connected to FAD scarcity (or a temporary FMN/Rf accumulation, due to FADS activity slowed down).

Regulation of Rf transporters by Rf concentration was reported both in *C. elegans* ^27^ and in human intestine, the latter involving the transcriptional regulator Sp1 controls.^43^ Human Sp1 transcription factor can, in turn, be regulated by stress induced by TNF‐α, which has an inhibitory effect on Rf uptake.^44^ Maybe a putative orthologue of Sp1 in *C. elegans* (*sptf‐2*, 64% sequence identity), expressed in head neurons and intestine, might sense Rf variations. Another hypothesis is that *rft‐1* changes are secondarily connected to ETFDH deficiency producing oxidative stress. Indeed, a secondary derangement of flavin cofactor homeostasis was recently demonstrated in a mammalian cell model in which a primary defect of ETFDH is causative of MADD.^45^

In humans, inborn errors of Rf transporters, that is, RTDs, are now well‐related to neurological dysfunction: they primarily affect neuronal mitochondrial flavoproteome functionality,^17^, ^46^ resulting in ROS unbalance and morphological alterations of the organelles.^47^ RTDs are also treatable with high doses of the vitamin and this suggests a direct or indirect role of flavins in regulating the expression of their own translocators. Therefore, a secondary neuronal phenotype in *flad‐1* silenced animals might be related to alteration of *rft‐1* transcript level.

Surely, interfered *C. elegans* clearly present locomotion impairments, which are cured by Rf and its derivatives. Defects in cholinergic transmission are demonstrated by the increased sensitivity to aldicarb, already observed in the N2 strain^21^ and even more evident in the *rrf‐3 (pk1426)* strain.

We propose here that a pre‐synaptic neuronal defect, linked to FAD decrease and secondary to acetylcholine scarcity, could be responsible for locomotion phenotype. A defect in neuro‐mediator synthesis/release, rather than muscular reception, was definitively assessed on the bases of a retained sensitivity to levamisole of both egg laying and locomotion behavior. The suggested neuronal neurotransmitter deprivation is, nevertheless, superimposable to beta‐oxidation impairments, predictably affecting muscular energy production, fertility, and egg laying.^23^

Consistently, in interfered animals, externally added acetylcholine produces beneficial effects similar to that of flavins on locomotion behavior.

In cholinergic neurons, acetyl‐CoA is specifically requested for the neuro‐mediator synthesis via the reaction catalysed by choline O‐acetyltransferase (EC 2.3.1.6). Since acetyl‐CoA fluxes from pyruvate and via beta‐oxidation and TCA cycle are metabolically strictly linked to mitochondrial flavoprotein activities,^2^ its level is expected to be altered by silencing *flad‐1*. On the other hand, the implication of FAD‐dependent enzymes in choline recycling pathways^48^, ^49^ cannot be, at the moment, ruled out. Therefore, unravelling the precise contribute of FAD availability to neurotransmitter “life” will be matter for further research.

While this study was in progress in the National Bioresource Project for the Experimental Animal “Animal *C. elegans*” (https://shigen.nig.ac.jp/c.elegans/) a stable *flad‐1* deficient strain was generated and now available as homozygous viable. This surely could facilitate experiments aimed at searching both alternative pharmacological strategy and the molecular bases for Rf therapy.

## CONFLICT OF INTEREST

The authors declare no potential conflict of interest.

## AUTHOR CONTRIBUTIONS

Piero Leone manuscript writing and immunoblot analysis. Maria Tolomeo quantitation of flavins and statistical analysis. Elisabetta Piancone under the supervision of Elia Di Schiavi *C. elegans* culture and RNA interference, chemosensory and egg‐production assays. Pier Giorgio Puzzovio under the supervision of Carla De Giorgi RT‐PCR experiments and bioinformatics. Cesare Indiveri critical revision of the experimental work and of the manuscript draft. Maria Barile coordination and supervision of the work.

**Abbreviations**
ACh: acetylcholine
ALS: amyotrophic lateral sclerosis
BVVLS: Brown–Vialetto–Van Laere syndrome
ETFDH: electron transfer flavoprotein dehydrogenase
FADS: FAD synthase
FAD: flavin adenine dinucleotide
FMN: flavin mononucleotide
LSMFLAD: lipid storage myopathy due to FAD synthase deficiency
MADD: multiple Acyl‐CoA dehydrogenase deficiency
MPTb: molybdoPterin‐binding
mtUPR: mitochondrial unfolding protein response
NGM: nematode growth medium
Rf: riboflavin
RFK: riboflavin kinase
RFVT: riboflavin transporter
ROS: reactive oxygen species
RTD: riboflavin transport deficiency
SLC: solute carrier
Sp1: stimulating protein 1
TCA: tricarboxylic acid
TNF‐α: tumor necrosis factor alpha

## Supporting information


**FIGURE S1** Transcript levels in *flad‐1* silenced *rrf‐3 (pk1426)* and N2 *C. elegans*. *flad‐1*, *let‐721*, *sdha‐1*, and *lsd‐1* transcript levels in *flad‐1* silenced *rrf‐3 (pk1426)* (A) and N2 (B) animals. The histogram panel was obtained by quantification of the cDNA bands as described in Experimental Procedure; each value is the mean ± SD of three different determinations (**p ≤ 0.01; ***p ≤ 0.001). *flad‐1*, *rft‐1*, and *rfk‐1* transcript levels in *flad‐1* silenced *rrf‐3 (pk1426)* (C) and N2 (D) animals. The histogram panel was obtained by quantification of the cDNA bands as described in Experimental Procedure; each value is the mean ± SD of three different determinations (**p ≤ 0.01)

**TABLE S1** Primers used for RT‐PCR experiments.
**TABLE S2**. Flavin content (nmol/mg protein) in control and silenced *rrf‐3 (pk1426)* nematodes and the percentage of reduction/increase with respect to the control are reported. Data represent the mean ± SD of three independent experiments
