# Supplementary figures and images for: Mimicking human riboflavin responsive neuromuscular disorders by silencing flad‐1 gene in C. elegans : Alteration of vitamin transport and cholinergic transmission
Source: IUBMB Life. 2021 Sep 24;74(7):672–83. doi: 10.1002/iub.2553 (PMC9292511; doi:10.1002/iub.2553)

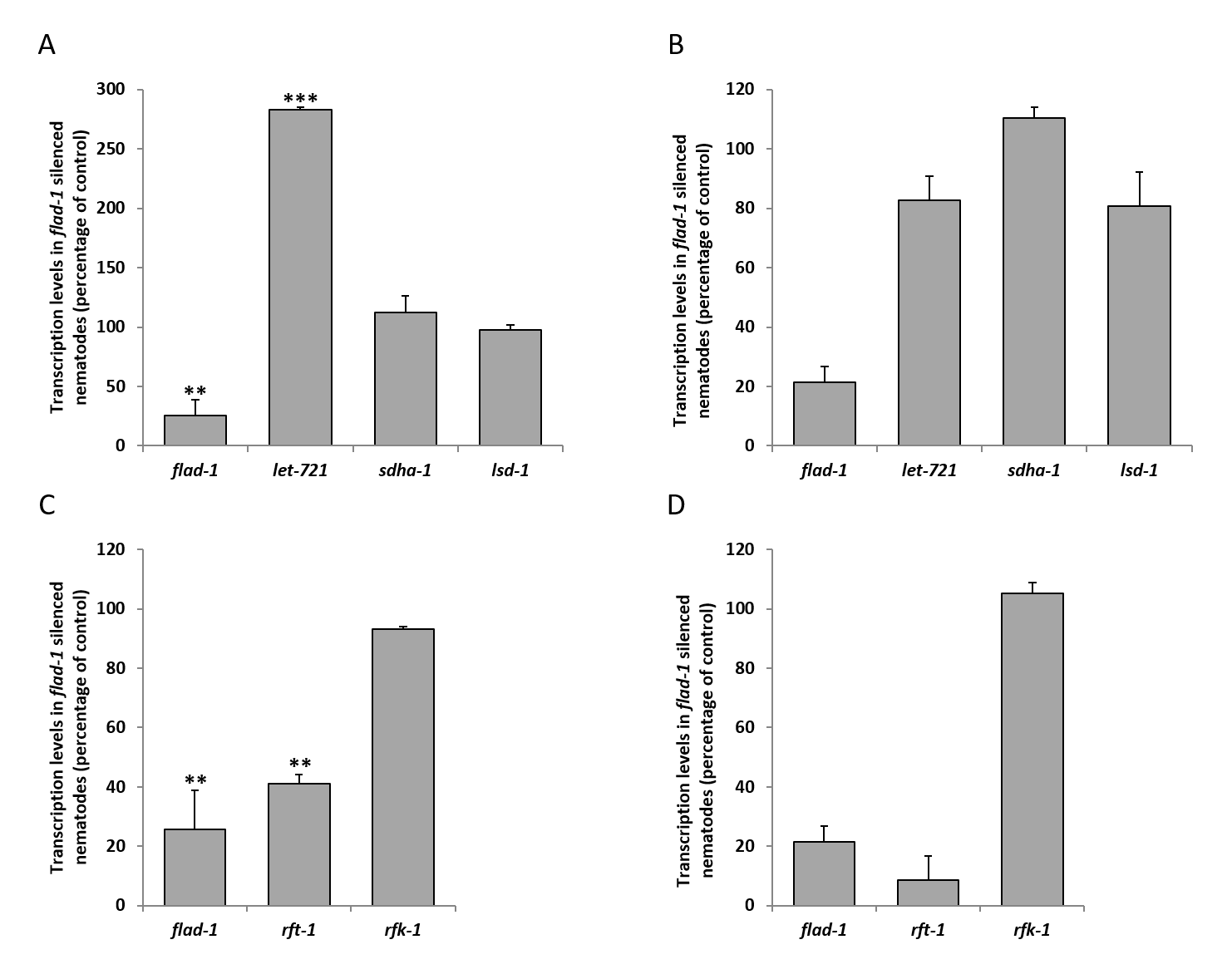

Supplement: Supplementary file 1 — FIGURE S1 Transcript levels in flad‐1 silenced rrf‐3 (pk1426) and N2 C. elegans. flad‐1, let‐721, sdha‐1, and lsd‐1 transcript levels in flad‐1 silenced rrf‐3 (pk1426) (A) and N2 (B) animals. The histogram panel was obtained by quantification of the cDNA bands as described in Experimental Procedure; each value is the mean ± SD of three different determinations (**p ≤ 0.01; ***p ≤ 0.001). flad‐1, rft‐1, and rfk‐1 transcript levels in flad‐1 silenced rrf‐3 (pk1426) (C) and N2 (D) animals. The histogram panel was obtained by quantification of the cDNA bands as described in Experimental Procedure; each value is the mean ± SD of three different determinations (**p ≤ 0.01) [file IUB-74-672-s001.tif]
