# Supplementary material for: Mimicking human riboflavin responsive neuromuscular disorders by silencing flad‐1 gene in C. elegans : Alteration of vitamin transport and cholinergic transmission
Source: IUBMB Life. 2021 Sep 24;74(7):672–83. doi: 10.1002/iub.2553 (PMC9292511; doi:10.1002/iub.2553)
Supplement: Supplementary file 2 — TABLE S1 Primers used for RT‐PCR experiments. TABLE S2. Flavin content (nmol/mg protein) in control and silenced rrf‐3 (pk1426) nematodes and the percentage of reduction/increase with respect to the control are reported. Data represent the mean ± SD of three independent experiments [file IUB-74-672-s002.docx]

**Supplementary Table 1.** Primers used for RT-PCR experiments.

| **Gene name** | **Primer FW (5’>3’)** | **Primer REV (5’>3’)** | **Annealing temperature (°C)** |
| --- | --- | --- | --- |
| *flad-1* | TCGGCGATGAGATTCTGAAG | CAACGTCTCGAAGCACAGAG | 55 |
| *ama-1* | TCAGCAGTTGCAGAGAAATGA | TTTTGTGGAGAGTCGGTTGAC | 55 |
| *let-721* | CACATTATTCCGCGTCTCAT | GTGCTCCATCCTTTCCAATT | 53 |
| *hsp-90* | AGAAGGAGCGTGAGAAGGAG | TTCTGGCATGAGCTCTTCGC | 53 |
| *sdha-1* | GGAGTTGGACCAGACAAGGA | AAGCTCGGCAGTTGAGATGT | 55 |
| *lsd-1* | CTAGGATCTATCAACGAGGAAG | AAACTTCTTCTCCATGTTGTTG | 53 |
| *rfk-1* | TGAATCTGTTACCCTACCAATT | GCAGATTTTAGTTCGTCTAGTG | 53 |
| *rft-1* | GGTGTTAGCATGTATTTGTC | GCGAAGAGCATAATGTTAGA | 51 |

**Supplementary Table 2.** Flavin content (nmol/mg protein) in control and silenced *rrf-3 (pk1426)* nematodes and the percentage of reduction/increase with respect to the control are reported. Data represent the mean ± SD of three independent experiments.

|  | **FAD** | **FMN** | **Rf** | **Total content of flavins** | **Percentage of control** |
| --- | --- | --- | --- | --- | --- |
| **CTR** | 0.83 ± 0.06 | 4.52 ± 0.01 | 1.24 ± 0.01 | 6.6 | - |
| **RNAi** | 0.47 ± 0.03 | 3.57 ± 0.02 | 0.64 ± 0.001 | 4.7 | - 28.9 % |
| **CTR+Rf** | 0.93 ± 0.05 | 8.81 ± 0.11 | 3.39 ± 0.11 | 13.2 | + 100.1 % |
| **RNAi+Rf** | 0.71 ± 0.01 | 7.70 ± 0.30 | 1.51 ± 0.04 | 9.9 | + 50.5 % |
| **CTR+FAD** | 0.72 ± 0.11 | 9.80 ± 0.13 | 4.78 ± 0.37 | 15.3 | + 132.1 % |
| **RNAi+FAD** | 0.71 ± 0.01 | 6.60 ± 0.38 | 1.54 ± 0.09 | 8.8 | + 34.2 % |
